# Targeted Delivery of Soluble Guanylate Cyclase (sGC) Activator Cinaciguat to Renal Mesangial Cells via Virus-Mimetic Nanoparticles Potentiates Anti-Fibrotic Effects by cGMP-Mediated Suppression of the TGF-β Pathway

**DOI:** 10.3390/ijms22052557

**Published:** 2021-03-04

**Authors:** Daniel Fleischmann, Manuela Harloff, Sara Maslanka Figueroa, Jens Schlossmann, Achim Goepferich

**Affiliations:** 1 Department of Pharmaceutical Technology, University of Regensburg, 93053 Regensburg, Germany; Daniel.Fleischmann@ur.de (D.F.); sara.maslanka-figueroa@ur.de (S.M.F.); 2 Department of Pharmacology and Toxicology, University of Regensburg, 93053 Regensburg, Germany; m_harloff@mailbox.org (M.H.); jens.schlossmann@ur.de (J.S.)

**Keywords:** sGC activators, cGMP, nanoparticle drug delivery, mesangial cells, diabetic nephropathy

## Abstract

Diabetic nephropathy (DN) ranks among the most detrimental long-term effects of diabetes, affecting more than 30% of all patients. Within the diseased kidney, intraglomerular mesangial cells play a key role in facilitating the pro-fibrotic turnover of extracellular matrix components and a progredient glomerular hyperproliferation. These pathological effects are in part caused by an impaired functionality of soluble guanylate cyclase (sGC) and a consequentially reduced synthesis of anti-fibrotic messenger 3′,5′-cyclic guanosine monophosphate (cGMP). Bay 58-2667 (cinaciguat) is able to re-activate defective sGC; however, the drug suffers from poor bioavailability and its systemic administration is linked to adverse events such as severe hypotension, which can hamper the therapeutic effect. In this study, cinaciguat was therefore efficiently encapsulated into virus-mimetic nanoparticles (NPs) that are able to specifically target renal mesangial cells and therefore increase the intracellular drug accumulation. NP-assisted drug delivery thereby increased in vitro potency of cinaciguat-induced sGC stabilization and activation, as well as the related downstream signaling 4- to 5-fold. Additionally, administration of drug-loaded NPs provided a considerable suppression of the non-canonical transforming growth factor β (TGF-β) signaling pathway and the resulting pro-fibrotic remodeling by 50–100%, making the system a promising tool for a more refined therapy of DN and other related kidney pathologies.

## 1. Introduction

With an estimated number of almost 500 million cases worldwide and an ever-growing prevalence throughout all age groups, diabetes ranks among the top global causes of death [1]. While the imminent consequences of a poor blood sugar homeostasis are minor, long-term diabetic complications severely limit the life expectancy of patients. Among these, chronic kidney disease (CKD) and eventual end-stage renal disease (ESRD), i.e., the ultimate loss of kidney function, are regarded to be most critical [2,3,4]. Within the plentiful concomitants of CKD, diabetic nephropathy (DN) plays a central role, affecting more than 30% of diabetes patients [5,6]. When it comes to DN, a plethora of pathological renal processes are initiated by a prolonged state of hyperglycemia and eventually culminate in a progredient decline in glomerular filtration capacity [7]. Among these processes, increasing podocyte damage and basement membrane thickening, as well as an increased glomerular hypertrophy and fibrosis have been found to play a substantial role [8,9]. In that context, the glomerular mesangium acts as a key player by facilitating a fundamentally altered production of extracellular matrix components, as well as a broad range of paracrine interactions with glomerular endothelial cells and podocytes [10,11,12]. Within the highly complex network of molecular mechanisms that are responsible for this pathological remodeling, progredient activation of transforming growth factor β (TGF-β) signal cascades have been shown to be a central element, especially regarding an increased extracellular matrix (ECM) remodeling and glomerular hyperproliferation [13,14,15]. However, there is also unanimous evidence that apart from other pathological triggers such as elevated oxidative stress or an enhanced activity of the renin angiotensin aldosterone system (RAAS), a dysfunctionality of nitric oxide (NO) and its downstream cascade plays a pivotal role [16,17,18]. Under physiological conditions, NO can bind the intracellularly located soluble form of the guanylate cyclase (sGC) that then transforms guanosine triphosphate (GTP) to 3′,5′-cyclic guanosine monophosphate (cGMP), a central messenger for the downregulation of excessive glomerular fibrosis and hyperproliferation [19,20,21]. Under hyperglycemic conditions, however, NO production is impaired due to a progrediently dysfunctional endothelium [22]. Additionally, the abovementioned hyperglycemia-induced oxidative stress can lead to an oxidation or even loss of the sGC heme prosthetic group (Fe^3+^-/Apo-sGC), making the enzyme unresponsive to any NO activation [23,24]. Initially, pharmacological research concerning the NO–cGMP cascade—particularly in the field of cardiovascular disease—was mainly focused on an increase of nitric oxide levels via NO-donating substances or an inhibition of cGMP-degrading phosphodiesterases [25]. However, the impact of these approaches, as well as sGC stimulators such as riociguat, relies on a sufficient residual activity of sGC and is therefore severely limited if an sGC heme oxidation or loss has already taken place. In contrast, sGC activators such as Bay 58-2667 (cinaciguat; CCG) are able to specifically bind and thus re-activate defective Fe^3+^-/Apo-sGC independent of the presence of NO. This characteristic provides the fundamental advantage that a therapeutic effect is also achievable in a more advanced stage of disease. Initial research on CCG was mainly focused on its therapeutic effects regarding the cardiovascular system [26]; however, there is also promising evidence concerning a beneficial role in the above-discussed renal fibrosis and hyperproliferation [27,28,29], whereby a CCG-mediated sGC activation has been shown to reduce TGF-β expression as well as non-canonical TGF-β signaling via extracellular-regulated protein kinase 1/2 (ERK 1/2) [30,31]. While these results were highly encouraging, CCG can however also potentially cause unfavorable adverse events, such as a strong hypotension, due to its imminent effect on the vasculature after a systemic administration [32]. The application of the free drug is additionally complicated by its profound lipophilicity, which could possibly hamper the therapeutic success due to a reduced solubility or a generally disadvantageous biodistribution [33]. To our estimation, CCG would therefore substantially profit from a drug delivery system that is able to considerably increase the drug accumulation in target regions, such as the above-discussed mesangium, while minimizing interactions with off-target sites, such as the vasculature.

When it comes to targeted drug delivery, nanoparticular approaches provide considerable benefit in that they enable the differentiation of targeted tissues from unfavorable off-target sites via functionalization with cell-selective recognition sequences for target cell surface structures [34,35]. Implementation of this principle already led to a great number of nanoparticular devices with a substantial specificity for target cells within various tissues, such as the colon, liver, or also the kidney [36,37,38].

**Figure 1:**
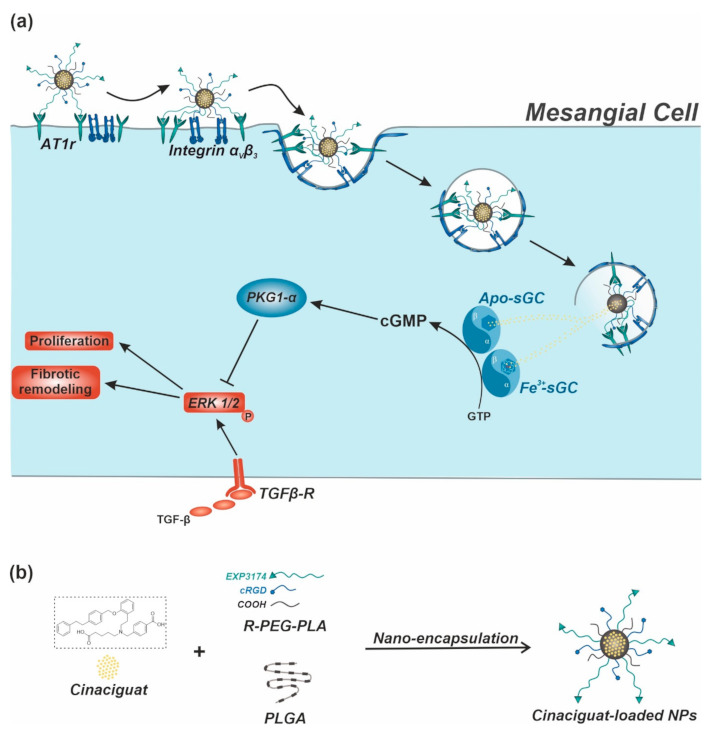
Therapeutic concept. (**a**) Nanoparticle (NP)-assisted cinaciguat (CCG) delivery to intracellular Apo-/Fe^3+^-sGC of target mesangial cells. After a sequential and thereby highly cell-selective mesangial NP uptake, CCG is released into the cytosol due to endolysosomal degradation of the NP. Here, CCG binds and thus activates both oxidized and heme-free sGC, leading to an increased production of 3′,5′-cyclic guanosine monophosphate (cGMP) and a protein kinase 1 α (PKG1-α)-mediated inhibition of transforming growth factor β (TGF-β)-induced pathological remodeling. (**b**) Manufacturing principle for CCG-loaded virus-mimetic NPs.

In that regard, we recently presented an actively targeted nanoparticle (NP) species that enabled a highly specific mesangial in vivo targeting within the kidney [39]. The NP system is based on a virus-mimetic presentation of two ligands that selectively recognize mesangial surface components and enable a reliable discrimination of mesangial sites and possible off-targets. Inspired by its biological model, human adenovirus type 2 [40], the NP thereby interacts with the cell surface in a sequential manner of initial binding to the angiotensin II receptor type 1 (AT1r) via an AT1r blocker (EXP3174), followed by a presentation of previously shielded cyclic amino acid sequence (cRGD) that activates mesangial surface integrin α_v_β_3_ and eventually triggers cell endocytosis (Figure 1a) [41].

As this NP system provided excellent mesangial in vivo specificity, we hypothesized that virus-mimetic NPs loaded with CCG (Figure 1b) could substantially increase drug delivery into intracellular compartments of target mesangial cells. After a subsequent processing and endolysosomal degradation of the NP system, the active substance would be released and then bind and thus stimulate cytosolic sGC, both in its oxidized and heme-free form, which would eventually lead to a significant rise in cGMP-mediated signaling (Figure 1a).

In this study, we therefore initially loaded our NP system with CCG and assessed whether a therapeutically relevant amount of drug could be encapsulated while still preserving the described active mesangial NP targeting functionalities. In order to subsequently test the pharmacological impact of the NP-mediated drug delivery, we compared the effect of both free and NP-encapsulated CGG on its target, as well as various further downstream elements of the sGC-cGMP cascade such as the cGMP-dependent activation of protein kinase 1 α (PKG1-α). Finally, we determined the anti-fibrotic potential of the system in a TGF-β-induced in vitro fibrosis model (Figure 1a).

## 2. Results and Discussion

### 2.1. Efficient and Reproducible Encapsulation of CCG into Targeted NPs

To enable a NP-mediated, targeted delivery of CCG to mesangial sites, we initially assessed whether a pharmacologically sufficient amount of drug could be encapsulated into our NP system. The NP itself thereby consisted of shell-forming poly(ethylene glycol)−poly(lactic acid) (PEG-PLA) block-copolymers carrying the above-described targeting functionalities, as well as poly(lactic-co-glycolic acid) (PLGA), which both stabilizes the NP core and can be used to encapsulate suitable active agents such as CCG (Figure 1b). In this regard, we recently presented a study discussing the necessary physicochemical properties of encapsulated compounds in order to achieve a satisfactory NP loading [42]. We therein found that, for a successful encapsulation, substances on the one hand need to possess a sufficient lipophilicity, and, on the other hand, the miscibility of encapsulated drug and core forming PLGA has to exceed a certain threshold in order to guarantee NP stability. With regard to these prerequisites, CCG was found to be a promising candidate for NP encapsulation, showing a substantial lipophilicity (logP ≈ 7) and a calculatorily determined high miscibility with PLGA. In that regard, the Flory–Huggins parameter for CCG and PLGA was found to be around 0.005, indicating that both components were excellently miscible [43].

**Figure 2:**
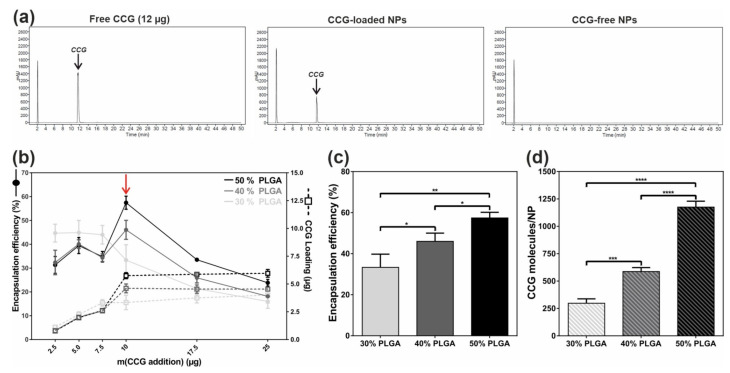
(**a**) Representative HPLC chromatograms of free CCG as well as CCG-loaded or drug-free NPs. CCG could thereby be reproducibly detected at a retention time of 12 min (black arrow). (**b**) CCG loading per NP batch and the respective encapsulation efficiency (EE) for NPs carrying either 30%, 40%, or 50% PLGA as core component. EE was thereby highest for an addition of 10 µG CCG per 4 mg NP batch (red arrow). (**c**) EE (10 µg CCG addition) gradually increased with larger amounts of PLGA added. (**d**) Calculated number of CCG molecules per NP type. (see also Figure S3a,b). Results represent mean ± SD (*n* = 3). * *p* < 0.05, ** *p* < 0.01, *** *p* < 0.001, **** *p* < 0.0001.

Based on these initial considerations, we decided to test three different NP compositions with a varying proportion of core PLGA (30/40/50%) to find an ideal compromise between high drug loading, satisfactory encapsulation efficiency, and small NP diameter. It was thereby particularly important that resulting NPs would not exceed 90–100 nm to still guarantee a sufficient in vivo extravasation through the fenestrated glomerular endothelium into mesangial areas [44]. For every NP composition, we then added rising levels of CCG to the polymer mix and—after nanoprecipitation and lyophilization of NPs—assessed the amount of encapsulated drug via high performance liquid chromatography (HPLC). Both free CCG and NP-derived CCG could thereby be reproducibly detected at a wavelength of 230 nm (Figure 2a and Figure S1).

As Figure 2b indicates, larger amounts of initially added drug led to a progredient rise in finally encapsulated CCG (CCG loading) for all three NP compositions, whereby NP quality (size/dispersity) still remained satisfactory (Figure S2a–c). However, an addition of more than 10 µg CCG per 4 mg NP batch did not produce any further rise in CCG loading but rather increased the tendency of NPs to form larger aggregates, indicating that the general NP loading capacity was exceeded (Figure S2d). For the following analysis, we therefore decided to add 10 µg of drug during nanoprecipitation to both enable a maximum CCG loading and preserve the NPs’ stability.

In a next step, we determined several physicochemical parameters to assess the optimal PLGA proportion per particle (for a more detailed description, please also refer to the Methods section, Figure S3a,b, as well as [42]). While the NPs’ hydrodynamic diameter was found to be below 100 nm for all three NP types (Figure S2c), the encapsulation efficiency was significantly rising with a greater proportion of PLGA in the NP core (Figure 2c). This was expected due to the greater volume of the NP core as well as the excellent miscibility of CCG and PLGA. To enable a more realistic assessment of the actual amount of encapsulated drug per NP, we then calculated the number of CCG molecules per NP (Figure 2d), whereby a similar trend of rising levels of encapsulated substance could be found. Based on the hydrodynamic diameter of each NP type, we then calculated the respective NP core volume and determined the number of CCG molecules that would ideally fit into one NP. Finally, this number was used to calculate the CCG entrapment efficiency, i.e., the ratio of the number of actually encapsulated and the ideally encapsulated CCG molecules. We thereby found a contrary trend for the tested NP compositions. Here, higher proportions of PLGA led to a generally decreasing entrapment efficiency, indicating that although NPs with a higher ratio of PLGA could generally encapsulate more drug, the actual rise in CCG molecule numbers was not as high as theoretically possible (Figure S3c). We therefore hypothesized that the general composition of our NP system might not allow any higher CCG loading due to its substantial lipophilicity and a possible destabilizing effect of greater drug concentrations in the NP core. However, we estimated. that the successfully encapsulated amount of drug was already sufficient to enable a substantial pharmacological effect due to the considerable mesangial targeting capacity and the resulting drug delivery impact of the NP system.

We finally chose to proceed with the NP species consisting of 40% PLGA and 60% PEG-PLA, as this NP type showed a satisfactory encapsulation efficiency (~46%) and CCG loading (~600 CCG molecules per NP) while still possessing an ideal diameter of around 80 nm.

As we were able to reproducibly encapsulate a considerable amount of CCG into our NP system, we next intended to assess whether these CCG-loaded NPs could still harness their attached ligands to enter intracellular regions of target mesangial cells. As described above, NP cell entry is thereby based on an initial AT1r binding and a subsequent presentation of previously shielded cRGD functionalities, which enable an integrin-mediated endocytosis. We therefore used a rat-derived mesangial cell line (rMCs), which showed a substantial expression of both targets (AT1r/α_v_β_3_ integrin), as our previous studies had already confirmed [39].

**Figure 3:**
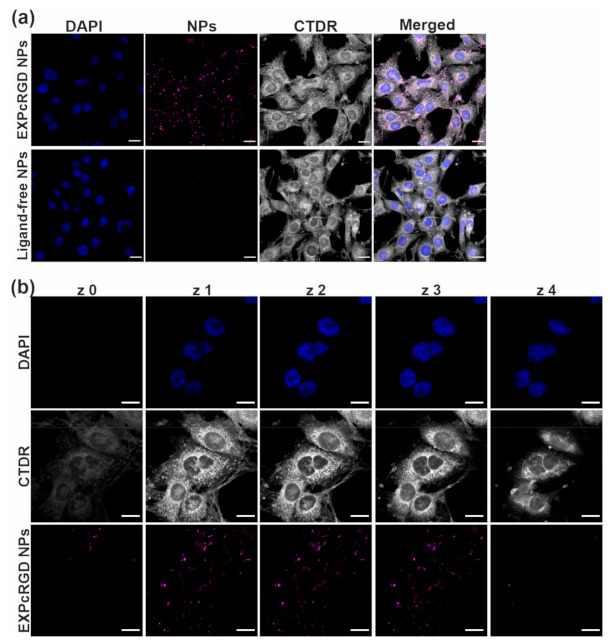
(**a**) CLSM analysis of CCG-loaded NP-cell interaction. In contrast to ligand-free control NPs, EXP3174/cRGD -targeted EXPcRGD NPs (purple) reached a substantial uptake into endocytotic vesicles of mesangial cells (cell cytosol = grey; cell nuclei = blue). (**b**) z-stack analysis further confirmed the intracellular localization of targeted NPs (EXPcRGD NPs) (z0–z4 = bottom to top z-plane). Scale bar = 20 µM.

In a first step, rMCs were incubated for 90 min with CCG-loaded NPs (10 µg per NP batch). The particles either carried both active ligands (EXPcRGD NPs) or were ligand-free control NPs. To enable a better visualization of particle uptake, a fluorescent label was covalently coupled to the PLGA prior to particle preparation, and cell cytosol was stained using CellTracker^®^ Deep Red dye (CTDR). As depicted in Figure 3a, ligand-functionalized EXPcRGD NPs showed a substantial mesangial accumulation, which corresponded to our previous results. In contrast, no visible cellular uptake could be detected for ligand-free control NPs, indicating that the mesangial cell uptake of EXPcRGD NPs was mediated via described ligand–receptor interactions. However, as the CCG target sGC is located intracellularly, we finally also assessed the exact location of visible NP accumulations to further confirm NP endocytosis and exclude a mere binding to the cell surface. We therefore performed z-stack analysis of rMCs after 90 min of EXPcRGD NP incubation and found that NP-associated fluorescence could almost entirely be found in sections representing intracellular segments of the cell body (Figure 3b). These observations were in line with our previous studies, which showed a maximum mesangial NP endocytosis after 90–120 min.

Taken together, these results confirmed our hypothesis that the active agent CCG can be efficiently transported into intracellular regions of mesangial cells via encapsulation in adenovirus-mimetic EXPcRGD NPs. The encapsulation process was thereby satisfactory and reproducible and did not interfere with the NPs’ ability to initially bind the AT1r via EXP3174 and subsequently initiate α_v_β_3_ mediated cell endocytosis.

### 2.2. Targeted CCG Delivery Potentiates sGC Activation and Stabilization

Having confirmed that actively targeted EXPcRGD NPs can be loaded with a pharmacologically relevant amount of CCG, our next goal was to investigate whether a more efficient transport of the active agent into the mesangial cytosol could possibly increase the drug’s potency due to a drug delivery effect. This hypothesis was based on the general assumption that the cellular uptake and cytosolic accumulation of free CCG might be limited, partly because of its substantial lipophilicity and the resulting poor solubility in aqueous systems and because of a merely passive diffusion into the target cell, which requires a constant concentration gradient. In contrast, CCG-loaded EXPcRGD NPs would be able to enter intracellular compartments more efficiently and release the active agent due to an endolysosomal degradation of the NP structure [45,46]. Additionally, the active targeting functionalities would enable a more selective in vivo transport of EXPcRGD NPs—and therefore also CCG—to mesangial sites within the kidney. This observation was already shown in our previous publication, where EXPcRGD NPs showed a considerably increased mesangial accumulation compared to ligand-free NPs [39].

**Figure 4:**
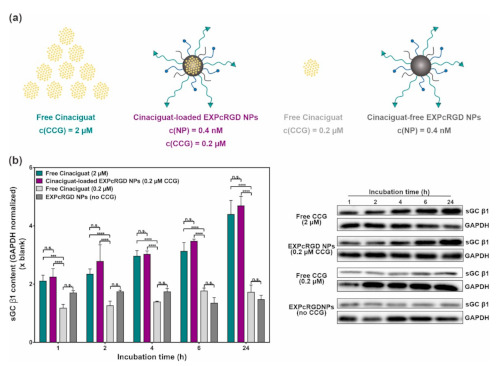
(**a**) Experimental set-up. In contrast to free CCG (c = 2 µM), drug-loaded EXPcRGD NPs carried merely 10% of the CCG dose to assess a possible drug delivery effect. (**b**) Western blot analysis of sGC β1 content upon sample incubation over 24 h showed significantly increased sGC β1 levels both for free CCG (2 µM) and the NP-encapsulated drug (0.2 µM), compared to a lower concentration of free CCG (0.2 µM) or drug-free NPs. Results represent mean ± SD (*n* = 3). *** *p* < 0.001, **** *p* < 0.0001, n.s., not significant.

To test the above-described drug delivery effect, we therefore decided to compare four major groups (Figure 4a). First, free CCG was administered at a concentration of 2 µM. This concentration was chosen as in previous studies it had been shown to exhibit a satisfactory and reproducible activation of the sGC cascade, which also led to an anti-fibrotic effect [31]. The second group consisted of CCG-loaded EXPcRGD NPs at a concentration of 0.4 nM, which on the one hand was comparable to our previous in vitro studies, whereby no cell-toxic effects had been observed [39], and on the other hand contained CCG at approximately 10% of the free dose (0.2 µM vs. 2 µM). By administering merely 1/10 of the free CCG dose, we wanted to test whether the encapsulation in targeted NPs could increase the CCG potency as described above and eventually provide comparable effects with fewer drug needed. As a third group, we therefore also applied free CCG at a lower concentration of 0.2 µM, which was similar to the NP-delivered amount of drug and should theoretically have provided a merely limited effect compared to the free drug at 2 µM [31]. Finally, CCG-free EXPcRGD NPs were administered in the same concentration as CCG-loaded NPs to exclude any possible impact of the NP-cell or ligand–receptor binding on the CCG effect.

We initially incubated rMCs with each of the four groups for 1 to 24 h and subsequently analyzed the sGC β1 content. Although CCG mainly acts as an activator of oxidized/heme-free sGC, previous studies indicated that due to a stabilizing effect, CCG-bound sGC can also be prevented from undergoing proteasomal degradation [47,48,49]. In our case, a corresponding trend could be observed, as shown in Figure 4b: Incubation with 2 µM of free CCG led to a substantial increase in sGC β1 content over 24 h. Interestingly, CCG-loaded EXPcRGD NPs provided a comparable effect, even though here, merely 10% of drug was administered in total. In contrast, free CCG at a similarly low concentration of 0.2 µM only showed a slight increase in sGC β1 levels, supporting our hypothesis that the NP-mediated CCG delivery can potentiate the pharmacological potency of the active substance. In this context, a possible impact of ligand–receptor or NP-cell interactions was negligible, as incubation with CCG-free NPs did not lead to an increase in sGC β1 content but rather showed a constant or slightly declining trend.

**Figure 5:**
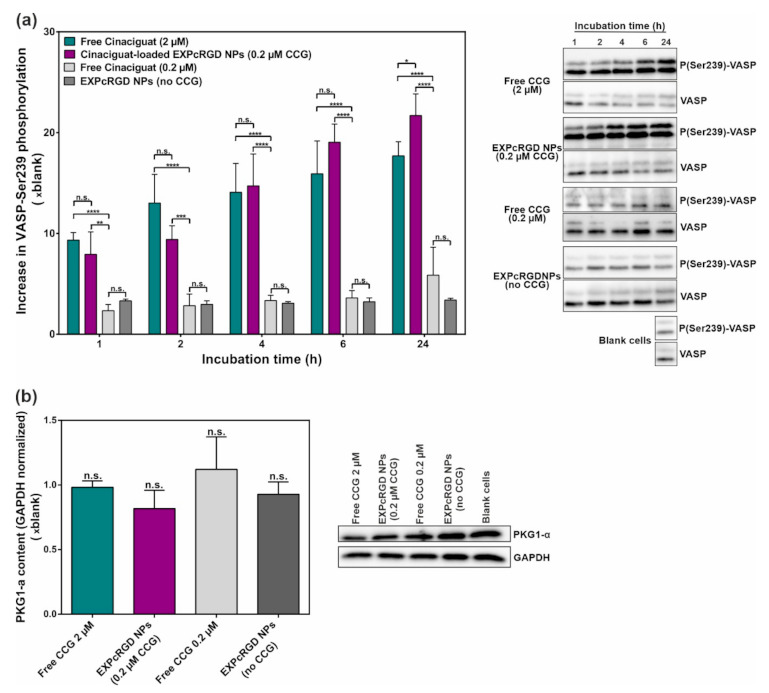
(**a**) Phosphorylation of PKG1-α substrate vasodilator-stimulated phosphoprotein (VASP)-serine 239 (Ser239) significantly increased over 24 h, both for free CCG (2 µM) and CCG-loaded EXPcRGD NPs (0.2 µM), indicating a substantial activation of the kinase. (**b**) Western blot (WB) analysis of total PKG1-α levels after 24 h incubation showed no considerable changes in protein content for any sample. Results represent mean ± SD (*n* = 3). * *p* < 0.05, ** *p* < 0.01, *** *p* < 0.001, **** *p* < 0.0001, n.s., not significant.

Since the main anti-fibrotic effect of sGC activators such as CCG is mediated via an eventual cGMP-derived activation of PKG1-α, we consequently also analyzed the respective impact of administered CCG/NPs on this kinase. In order to determine changes in activity, we chose to monitor the level of PKG1-α mediated phosphorylation of vasodilator-stimulated phosphoprotein (VASP) at serine 239 (P-VASP) [50]. As shown in Figure 5a, VASP phosphorylation thereby increased substantially over 24 h of incubation, both for free CCG (2 µM) and CCG-loaded NPs, which again carried merely 10% of the free CCG dose. Interestingly, this trend was initially higher for the free drug while at later time points, CCG-loaded NPs had a significantly increased effect on PKG1-α activity. To our estimation, this observation can be explained by the endolysosomal degradation that CCG-carrying NPs initially had to undergo until CCG was released into the cytosol. Again, incubation with a lower concentration of free CCG (0.2 µM) did not produce any noteworthy changes in VASP phosphorylation, even though here, the same amount of drug was applied as with the NP delivery system. Additionally, CCG-free NPs did not provide any changes in P-VASP/VASP ratio. To confirm that the observed upregulation of VASP phosphorylation was mediated via an enhanced PKG1-α activity, we additionally analyzed the PKG1-α content (Figure 5b). Here, no significant differences between all examined groups could be found, supporting the hypothesis of a cGMP-mediated activation of PKG1-α.

The depicted results clearly confirmed our initial hypothesis that CCG-loaded NPs can potentiate the agent’s pharmacological effect on sGC and its downstream cascade. The experienced sGC β1 activation/stabilization as well as the increase in PKG1-α activity was thereby comparable to the effect of the free drug, even though EXPcRGD NPs carried merely 10% of the dose. Remarkably, the free administration of the same CCG amount as in the NP system (0.2 µM) provided a considerably reduced pharmacological effect. In our view, this observation indicated that highly lipophilic drugs such as CCG can substantially profit from a more efficient delivery into intracellular regions of their target cells and thereby reach a comparable therapeutic effect in a significantly reduced concentration range.

### 2.3. CCG-Loaded NPs Show A Substantial Anti-Fibrotic Effect via Suppression of the Non-Canonical TGF-β Pathway

**Figure 6:**
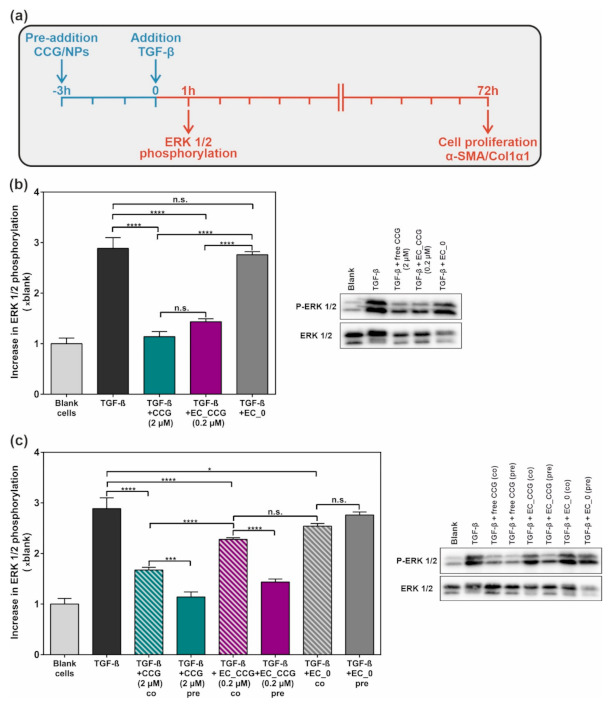
(**a**) In order to assess the anti-fibrotic potential of CCG-loaded NPs, a TGF-β based fibrosis model was established, whereby fibrotic key elements were analyzed at different time points. (**b**) Incubation of rat-derived mesangial cells (rMCs) with free CCG (2 µM) or CCG-loaded EXPcRGD NPs (EC_CCG; 0.2 µM, 10% of the free drug dose) prior to TGF-β exposure significantly reduced extracellular-regulated protein kinase 1/2 (ERK1/2) phosphorylation compared to CCG-free NP species (EC_0). (**c**) In contrast to a pre-incubation (“pre”), co-addition (“co”) of CCG/NPs and TGF-β led to a considerably lower reduction in ERK1/2 phosphorylation, especially for CCG-loaded NPs. Results represent mean ± SD (*n* = 3). * *p* < 0.05, *** *p* < 0.001, **** *p* < 0.0001, n.s., not significant.

As the NP-mediated CCG delivery led to a significantly potentiated activation of the sGC cascade, we lastly determined the anti-fibrotic potential of the system. In this context, previous studies had suggested that CCG-mediated sGC activation could be harnessed to substantially alleviate glomerular pathologies caused by elevated levels of TGF-β-associated fibrosis [30,31]. We therefore implemented a TGF-β-based in vitro fibrosis model and analyzed the therapeutic potential of free CCG (2 µM) as well as CCG-loaded EXPcRGD NPs (0.2 µM) (Figure 6a), as these two samples had provided a sufficient activation of the sGC–cGMP cascade, as described above. Again, drug-free EXPcRGD NPs served as a control to exclude any possible impact of the ligand–receptor or NP–cell interaction. Prior to cell incubation with described samples, rMC were starved from fetal bovine serum (FBS) for 24 h, as a significant amount of TGF-β is also present in FBS and could therefore have an impact on the later observed results.

Our first point of interest lay in ERK1/2, as previous studies had already indicated a substantial CCG-mediated suppression of the non-canonical TGF-β signaling pathway [31]. After 3 h of incubation with either free CCG (2 µM) or CCG-loaded EXPcRGD NPs (0.2 µM CCG), rMCs were therefore exposed to 10 ng mL^−1^ of TGF-β for 1 h and ERK1/2 phosphorylation was determined. Both for the free drug and CCG-loaded NPs, levels of P-ERK1/2 were thereby significantly reduced compared to a TGF-β control and CCG-free NPs. Similar to the experiments depicted in Figure 4; Figure 5, EXPcRGD NPs carried merely 10% of the drug dose but provided a comparable result, thereby further supporting our initial hypothesis of a drug delivery effect. We additionally tested the impact of a pre-incubation on the inhibitory effect by adding CCG/NPs as well as TGF-β at the same time-point (Figure 6c). Here, reduction of ERK1/2 phosphorylation was significantly lower compared to the pre-incubation. This trend was most noticeable for CCG-loaded EXPcRGD NPs, where only a minor inhibitory effect could be detected. To our estimation, these results indicated that the CCG-mediated effect was only possible after a successful intracellular uptake and degradation of NPs, thereby proving our hypothesized route of CCG delivery.

**Figure 7:**
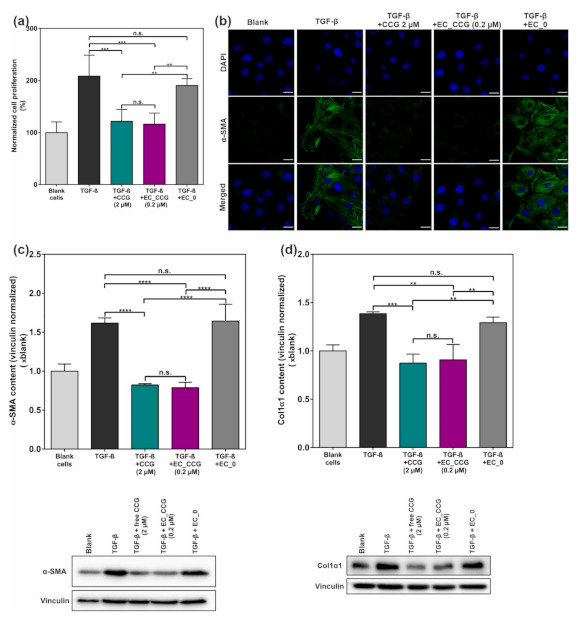
(**a**) A 3-(4,5-dimethylthiazol-2-yl)-2,5-diphenyltetrazolium bromide (MTT) cell proliferation assay revealed a significant reduction of TGF-β induced hyperproliferation by pre-incubation with CCG-loaded EXPcRGD NPs. (**b**) CLSM analysis of α-smooth muscle actin (α-SMA) expression showed a considerable reduction of pro-fibrotic α-SMA (green) for free CCG as well as CCG-carrying NPs. Cell nuclei = blue. Scale bar = 20 µM. WB analysis of (**c**) α-SMA and (**d**) Collagen 1α1 (Col1α1) expression proved the observation of a considerable anti-fibrotic potential of CCG-loaded EXPcRGD NPs. Results represent mean ± SD (*n* = 3). ** *p* < 0.01, *** *p* < 0.001, **** *p* < 0.0001, n.s., not significant.

As another key element of a TGF-β-associated remodeling is a substantial hyperproliferation of tissues, we next investigated the effect of our NP system on a possible reduction of cell proliferation. We therefore incubated rMCs with CCG/NPs as well as TGF-β similar to the experiments above and after 72 h performed a 3-(4,5-dimethylthiazol-2-yl)-2,5-diphenyltetrazolium bromide (MTT) proliferation assay. As depicted in Figure 7a, TGF-β induced hyperproliferation could thereby almost entirely be suppressed both with free CCG and EXPcRGD NPs, again carrying merely 10% of the free CCG dose. Drug-free NPs, in contrast, did not provide any significant anti-proliferative or cell-toxic effect. In this context, we also assessed a possible effect of the above-described serum starvation on the general cell viability of rMCs. As expected, deprivation of cells from FBS thereby led to a slight decrease in overall cell proliferation. However, no cell toxicity such as elevated levels of apoptosis could be observed, confirming the applicability of the model (Figure S4) in our set-up.

In a final step, we determined the inhibitory potential of the system on the expression of α-SMA as well as Col1α1, two key elements of a TGF-β-derived, myofibroblast-like differentiation of cells. Antibody staining and confocal laser scanning microscopy (CLSM) analysis of α-SMA content thereby revealed a trend, which was similar to the above-described effects (Figure 7b): While stimulation of rMCs with TGF-β led to a considerable increase in α-SMA signal within the cell body of rMCs, pre-incubation with EXPcRGD NPs carrying CCG substantially suppressed this effect, especially compared to CCG-free NPs. These observations were further supported by Western blot analysis of α-SMA content, where a similar anti-fibrotic effect was noticeable (Figure 7c). Accordingly, pre-incubation with CCG-loaded EXPcRGD NPs also significantly reduced expression of Col1α1 (Figure 7d). Again, the observed effect was comparable to the application of 10 times as much free drug.

Taken together, we suggest that the presented results confirm our initial hypothesis of a potentiated CCG effect due to a NP-derived drug delivery. Compared to the administration of the free drug, CCG-loaded NPs thereby showed a similar activation of the sGC pathway and suppression of the non-canonical TGF-β pathway, even though they carried merely 10% of the dose. To our estimation, this effect was mainly based on the fact that EXPcRGD NPs could transport their cargo more efficiently into cytosolic compartments of the target cell in contrast to the highly lipophilic free drug.

## 3. Materials and Methods

### 3.1. Materials

BAY 58-2667 (cinaciguat hydrochloride) was purchased from Axon Medchem BV (Groningen, The Netherlands). For NP preparation, heterobifunctional poly(ethylene glycol) components were obtained from Jenkem Technology USA Inc. (Allen, TX, USA), while methoxy poly(ethylene glycol) and Resomer RG 502 (PLGA) were purchased from Sigma-Aldrich (Taufkirchen, Germany). EXP3174 (losartan carboxylic acid) was obtained from Santa Cruz (Heidelberg, Germany), and cyclic RGDfK (cRGD) was purchased from Synpeptide Co. Ltd. (Shanghai, China). AlexaFluor 568 Hydrazide (Alexa568) and CellTracker Deep Red Dye (CTDR) were purchased from Fisher Scientific GmbH (Schwerte, Germany). For Lowry protein determination and later Western blot analysis, DC protein assay and Clarity™ Western ECL Blotting substrate were obtained from Bio-Rad Laboratories GmbH (Munich, Germany). Immobilon^®^ PVDF membranes were purchased from Millipore GmbH (Schwalbach, Germany). For Western blot analysis, the following primary antibodies and dilutions were used: sGC β1 (ER19), rabbit, 1:500 (Sigma-Aldrich); phospho-VASP (Ser239), rabbit, 1:1000; VASP (9A2), rabbit, 1:1000; phospho-p44/42 MAPK (p-ERK1/2), rabbit, 1:1000; p44/42 MAPK (ERK1/2), rabbit, 1:1000; GAPDH, rabbit, 1:1000 (all from Cell Signaling Technology, Cambridge, UK); PKG1-α, rabbit, 1:500 (own production [51]); Col1α1, rabbit, 1:500; α-SMA, mouse, 1:500 (Abcam, Cambridge, UK); vinculin, mouse, 1:500 (R&D Systems, Wiesbaden-Nordenstadt, Germany). Secondary antibodies: mouse-IgG horse radish peroxidase-conjugated, 1:10000 (Sigma-Aldrich); rabbit-IgG horse radish peroxidase-conjugated, 1:25,000 (Dianova GmbH, Hamburg, Germany). Transforming growth factor-β1 human (TGF-β) as well as all other utilized chemicals were purchased from Sigma-Aldrich in analytical grade if not stated differently. Immortalized, rat-derived mesangial cells (rMCs) were a kind gift from Prof. Dr. Armin Kurtz (Institute of Physiology, University of Regensburg, Regensburg, Germany). rMCs were isolated and cultured in RPMI 1640 medium containing 10% fetal bovine serum, insulin-transferrin-selenium (ITS) (1×), and 100 nM hydrocortisone as previously described [52,53].

### 3.2. Methods

#### 3.2.1. Synthesis and Functionalization of NP Polymer Components

COOH-/NH_2_-PEG_2k/5k_-PLA_10k_ block copolymers were synthesized and functionalized with EXP3174/cRGDfK as previously published by our working group [39,41,54,55,56], yielding longer EXP3174-PEG_5k_-PLA_10k_ and shorter cRGD-PEG_2k_-PLA_10k_. When needed, core component PLGA was additionally coupled to Alexa568 as described before to enable fluorescence imaging of NPs [39].

#### 3.2.2. NP Manufacture and CCG Encapsulation

Using the above-mentioned unfunctionalized/ligand-carrying block copolymers and core-forming PLGA, EXPcRGD NPs as well as ligand-free control NPs were manufactured via a well-established nanoprecipitation method [39,55,56]. In brief, all polymeric components were mixed in acetonitrile (ACN), whereby different ratios of PEG-PLA/PLGA (70/30; 60/40; 50/50) were prepared to test the ideal composition for CCG encapsulation. For ligand-carrying EXPcRGD NPs, the PEG-PLA proportion thereby consisted of 25% EXP3174-PEG_5k_-PLA_10k_, 15% cRGD-PEG_2k_-PLA_10k_, and 60% COOH-PEG_2k_-PLA_10k_ (ligand-free NPs: 100% COOH-PEG_2k_-PLA_10k_), which had previously been found to be ideal for the desired stepwise targeting concept [39]. The polymer mix was finally added dropwise to vigorously stirring millipore water (mpH_2_O), yielding the above-described NP species.

For drug encapsulation, a stock solution of 2 mg mL^−1^ CCG in a mixture of ACN/dimethyl sulfoxide (DMSO) (21/1; *v*/*v*) was prepared, and rising volumes of the CCG solution was added to the polymer mix prior to nanoprecipitation. The NP solution was stirred for 3 h at room temperature (RT) to remove all organic solvents and subsequently purified from non-encapsulated CCG via centrifugation using Pall Microsep filters (molecular weight cut-off, 30 kDa; Pall Corporation, NY, USA). The NPs were thereby centrifuged three times (1250× *g*; 10 min) and washed with mpH_2_O after every centrifugation step.

#### 3.2.3. NP Characterization

Manufactured NPs were analyzed regarding their hydrodynamic diameter with a Malvern Zetasizer Nano ZS (Malvern, Herrenberg, Germany). For a more thorough discussion of these aspects and the surface density of EXP3174 and cRGDfK on the NP surface, please refer to our previous publications [39,41].

#### 3.2.4. HPLC Analysis of Drug Encapsulation

For a detailed analysis of CCG encapsulation, HPLC analysis was performed using a PLRP-S column (Agilent Technologies Inc., Santa Clara, CA, USA) at 40 °C. The mobile phase consisted of a 10 mM sodium phosphate buffered solution (pH 7.4; A) and ACN (B) (gradient: 0 min 95% A/5% B; 9 min 65% A/35% B; 25 min 20% A/80% B; 45 min 20% A/80% B; 50 min 95% A/5% B), eluted at 1.0 mL min^−1^ [57]. CCG was detected at a wavelength of 230 nm (retention time = 12 min). For calibration, CCG dilutions of 0.0125–0.8 mg mL^−1^ in DMSO were prepared, injected in the HPLC system, and the resulting peak area was analyzed (injection volume = 15 µL). To investigate drug encapsulation efficiency for the different NP types, freshly prepared particles were freeze-dried for 72 h, weighed, and subsequently dissolved in DMSO and injected into the HPLC system (injection volume = 15 µL), yielding a CCG peak at the same retention time (Figure 2a).

#### 3.2.5. Calculatory Determination of Encapsulation Parameters

For a detailed explanation of the calculation of the Flory–Huggins parameter, please refer to our previous publications [42,43]. The encapsulation efficiency (*EE*) was calculated as depicted in Equation (1), whereby *m_e_* is the mass of encapsulated drug, and *m_a_* is the mass of initially added CCG.

(1) $$EE\;\left(\%\right)=\frac{m_{e}}{m_{a}}\;\times \;100$$

For the lyophilized NP batches, we then assessed the amount of NPs (*n_NP_*) using Equation (2), whereby *m_NP_* is the gravimetrically determined mass of the NP batch, *ρ*_(*NP*)_ is the estimated density of the NPs (1.25 g cm^−3^) [42], *d_h_* is the experimentally determined hydrodynamic diameter, and *N_A_* is the Avogadro constant. [54]

(2) $$n_{NP}=\frac{m_{NP}}{\rho_{\left(NP\right)}\frac{4}{3}\pi \;{\left(\frac{d_{h}}{2}\right)}^{3}}\;\times \;\frac{1}{N_{A}}$$

Next, the amount of CCG molecules per batch (*n_CCG_*) was calculated from the HPLC results, and the number of CCG molecules per NP (*N_exp_*) was determined with Equation (3).

(3) $$N_{exp}=\frac{n_{CCG}}{n_{NP}}$$

We then calculated the theoretical volume of the NP core (*V_core_*) for every NP sample as described before. [42] Assuming a maximum packing efficiency of 90% [58] as well as the molecular volume of CCG (*V_CCG_* = 0.725 nm^3^) [59], we then calculated the ideal number of CCG molecules per NP (*N_ideal_*) using Equation (4).

(4) $$N_{ideal}=\;\frac{V_{core}}{V_{CCG}}\;\times \;0.9$$

Finally, the CCG entrapment efficiency (*E_ET_*) was determined using the experimentally determined *N_exp_* and Equation (5).

(5) $$E_{ET}\left(\%\right)=\frac{N_{exp}}{N_{ideal}}\;\times \;100$$

#### 3.2.6. CLSM Analysis of Uptake

To confirm ligand-mediated NP uptake, target rMCs were initially stained with CTDR for 45 min (25 µM in serum-free medium, 37 °C, 5% CO_2_), subsequently seeded into 8-well slides (Ibidi, Gräfelfing, Germany) at a density of 8000 well^−1^ and incubated for 48 h at 37 °C and 5% CO_2_. CCG-loaded EXPcRGD NPs as well as ligand-free particles were prepared as described above—however, with Alexa 568-labeled PLGA (40% PLGA; 10 µg CCG addition per batch). Resulting NPs were adjusted to a concentration of 0.4 nM using Leibovitz’s buffer supplemented with 0.1% bovine serum albumin (BSA) and added to the cells for 90 min. After, cells were washed with Dulbecco’s phosphate buffered saline (DPBS) and fixed with 4% paraformaldehyde (PFA) in DPBS for 10 min. To enable visualization of the cell nuclei, cells were thereafter stained for 5 min with a 1:200 dilution of 4′,6-diamidine-2′-phenylindole dihydrochloride (DAPI) in DPBS. Final samples were subsequently analyzed at a Zeiss LSM 710 (Carl Zeiss Microscopy GmbH, Jena, Germany).

#### 3.2.7. Analysis of CCG Effect on Intracellular Targets

To study the effect of free and NP-encapsulated cinaciguat on mesangial intracellular targets, cells were seeded into 6-well plates (Corning Inc., Corning, NY, USA) at a density of 200,000 cells well^−1^ and grown for 48 h (37 °C, 5% CO_2_). Subsequently, cells were incubated for 1–24 h with either free CCG (c = 0.2/2 µM), CCG-loaded EXPcRGD NPs (c(NP) = 0.4 nM; c(CCG) = 0.2 µM), or CCG-free EXPcRGD NPs (c(NP) = 0.4 nM). All samples were thereby adjusted to the specified concentrations using serum-free RPMI 1640 medium. After incubation, samples were aspirated, and cells were processed as previously described [60]. In brief, cells were washed with DPBS and harvested by adding 80 µL of lysis buffer (2% Lubrol, 20 mM Tris, 150 mM NaCl; protease inhibitors: 0.5 µg mL^−1^ leupeptin, 1 mM benzamidine, 0.3 mM phenylmethylsulfonyl fluoride) and using a cell scraper. Cells were thereafter homogenized and centrifuged for 15 min at 16,500× *g* and 4 °C to remove cell remnants. For the following determination of the protein concentration of cell lysates, a modified method of Lowry was used [61]. Cell samples were thereafter denatured, separated using a 11.5% SDS-PAGE (30 µg protein lane^−1^), and Western blotted using the above-described primary antibodies against sGC, (P-)VASP, PKG1-α, as well as GAPDH. Horseradish peroxidase coupled secondary antibodies was added, and, finally, activity was assessed after the addition of Clarity™ Western ECL Blotting substrate using a chemiluminescence detector (ChemiDoc MP System; Bio-Rad Laboratories GmbH, Munich, Germany) and ImageLab software [62]. Results for both sGC and PKG1-α were thereby normalized to the GAPDH content per sample. For VASP, the ratio of P-VASP and VASP was determined and normalized to the P-VASP/VASP ratio of untreated control cells.

#### 3.2.8. Analysis of Anti-Fibrotic CCG Effects

To determine the anti-fibrotic potential of CCG-loaded EXPcRGD NPs, we implemented a fibrosis model, whereby rMCs were initially seeded into 6-well plates as described above and serum-starved after 24 h in order to exclude any impact of serum-derived TGF-β [63]. After another 24 h, free CCG or CCG-carrying or CCG-free EXPcRGD NPs was added as described above. After incubation for 3 h, 10 ng mL^−1^ of TGF-β per well was added to induce pro-fibrotic changes. Additionally, for a second group, free CCG/NPs and TGF-β were added simultaneously. Cells were thereafter incubated for either 1 h (ERK1/2 phosphorylation) or 72 h (α-SMA/Col1α1 content), depending on the intended read-out (see also Figure 6a). After incubation, cells were harvested as described above, followed by SDS-PAGE and Western blot analysis with the respective antibodies. Resulting band intensities of α-SMA/Col1α1 were finally normalized to the respective vinculin content, while for ERK1/2, the ratio of P-ERK1/2 and ERK1/2 was determined and normalized to the P-ERK1/2/ERK1/2 ratio of untreated cells.

#### 3.2.9. Cell Proliferation Assay

To assess the impact of discussed samples on a TGF-β induced mesangial hyperproliferation, a 3-(4,5-dimethylthiazol-2-yl)-2,5-diphenyltetrazolium bromide (MTT) reduction assay was performed. In brief, rMCs were seeded into a 96-well plate (Corning Inc.) at a density of 3000 cells well^−1^ and incubated for 48 h, whereby cells were serum-starved after 24 h. Cells were thereafter incubated for 72 h with samples and TGF-β in serum-free RPMI 1640 medium as described in the previous section. After 72 h, samples were removed and 50 µL of RPMI medium containing 1 mg mL^−1^ MTT was added per well. After 3 h of incubation, the MTT solution was aspirated, and 100 µL of isopropanol was added for 30 min under light exclusion and gentle shaking. Finally, absorbance at 570 and 690 nm was assessed at a FluoStar Omega fluorescence microplate reader (BMG Labtech, Ortenberg, Germany). For cell viability, the difference in absorbance at 570 and 690 nm was determined. Results were finally normalized to untreated control cells.

#### 3.2.10. CLSM Analysis of α-SMA Expression

For visualization of changes in α-SMA deposition, rMCs were initially seeded into 8-well Ibidi slides as described above. After 24 h, cells were serum-starved, and after another 24 h, cells were incubated with either free CCG, CCG-loaded, or CCG-free EXPcRGD NPs for 3 h, followed by a TGF-β stimulation for 72 h (c = 10 ng mL^−1^) in serum-free RPMI 1640 medium. Cells were thereafter washed, fixed with 4% PFA for 10 min at RT, and permeabilized with 0.1% Triton-X in DPBS for 10 min at RT. After a washing step, unspecific binding sites were blocked by addition of a 1% solution of BSA in DPBS for 30 min. Subsequently, cells were incubated overnight with a mix of AlexaFluor^®^ 488-coupled antibody against α-SMA (Sigma-Aldrich) and DAPI (1:500 in 0.1% BSA in DPBS), followed by CLSM analysis.

#### 3.2.11. Statistical Analysis

Results are expressed as mean ± standard deviation (SD), whereby *n* represents technical replicates. Statistical parameters were calculated using GraphPad Prism 6 software [64]. One-way analysis of variance (ANOVA) as well as Tukey post-test were performed for calculation of statistical differences between analyzed groups. The resulting *p*-values are stated in each individual figure.

## 4. Conclusions

The above-discussed results of this study revealed three major outcomes. First, a pharmacologically relevant dose of CCG could be reproducibly encapsulated into our virus-mimetic NP system while still guaranteeing a highly efficient mesangial NP uptake due to the previously presented targeting functionalities. Second, NP-assisted delivery of CCG into the mesangial cytosol substantially potentiated the sGC activating effect of the drug. In this regard, NP-mediated transport of 10% of the original free dose led to a comparable pharmacological effect concerning sGC stabilization and activation. Remarkably, the same concentration did not provide any noteworthy effect when it was not delivered via the NP system but merely administered in its free form. As above mentioned, this outcome was possibly based on a more efficient and prolonged transport of NP-encapsulated CCG into cytosolic regions of the target cells. Lastly, activation of the sGC–cGMP cascade via CCG-loaded NPs led to a considerable reduction of non-canonical TGF-β signaling, resulting in significantly decreased levels of pro-fibrotic markers and a substantially reduced hyperproliferation. Again, the described effects could be realized with merely 10% of the free CCG dose, indicating the great potential of the NP system in the therapy of renal fibrosis and further pathologies. It would therefore be of utmost interest to test the described system in an in vivo disease model. In that regard, future studies would also have to elucidate whether glomerular damage cannot only be prevented but also be reversed by the administration of CCG-loaded NPs. As this effect has already been shown repeatedly for the free CCG drug or for sGC stimulators [29,30,31], we suppose a NP-mediated drug delivery could potentially even increase the observed therapeutic effect. In that regard, CCG transport via our virus-mimetic NP system would additionally provide the substantial advantage that it can prevent an imminent interaction of CCG with the vasculature and thereby minimize possible adverse effects such as a drop in blood pressure. When it comes to diabetes and the related kidney pathologies, this new treatment option would be highly beneficial as it would not only be able to prevent further kidney damage like the currently predominant therapy options (control of blood sugar/pressure) but would also have a substantial effect in the case of an already further progressed state of disease. In this regard, NP-assisted delivery of active substances such as CCG could be a decisive tool to increase target accumulation and drug bioavailability.

## Data Availability

The data presented in this study are available on request from the corresponding author. The data are not publicly available due to internal reasons.

## Supplementary Materials

The following are available online at https://www.mdpi.com/1422-0067/22/5/2557/s1.

## References

[1] Cho N., Shaw J.E., Karuranga S., Huang Y., da Rocha Fernandes J.D., Ohlrogge A.W., Malanda B. (2018). IDF Diabetes Atlas: Global revalence for 2017 and projections for 2045. Diabetes Res. Clin. Pract..

[2] Romagnani P., Remuzzi G., Glassock R., Levin A., Jager K.J., Tonelli M., Massy Z., Wanner C., Anders H.-J. (2017). Chronic kidney disease. Nat. Rev. Dis. Primers.

[3] Hill N.R., Fatoba S.T., Oke J.L., Hirst J.A., O’Callaghan C.A., Lasserson D.S., Hobbs F.D.R. (2016). Global Prevalence of Chronic Kidney—A Systematic Review and Meta-Analysis. PLoS ONE.

[4] Webster A.C., Nagler E.V., Morton R.L., Masson P. (2017). Chronic Kidney Disease. Lancet.

[5] Thomas M.C., Brownlee M., Susztak K., Sharma K., Jandeleit-Dahm K.A.M., Zoungas S., Rossing P., Groop P.-H., Cooper M.E. (2015). Diabetic kidney disease. Nat. Rev. Dis. Primers.

[6] Umanath K., Lewis J.B. (2018). Update on Diabetic Nephropathy: Core Curriculum 2018. Am. J. Kidney Dis..

[7] Magee C., Grieve D.J., Watson C.J., Brazil D.P. (2017). Diabetic Nephropathy: A Tangled Web to Unweave. Cardiovasc. Drugs Ther..

[8] Rao V., Tan S.H., Candasamy M., Bhattamisra S.K. (2019). Diabetic nephropathy: An update on pathogenesis and drug development. Diabetes Metab. Syndr..

[9] Kanwar Y.S., Sun L., Xie P., Liu F.-Y., Chen S. (2011). A glimpse of various pathogenetic mechanisms of diabetic nephropathy. Annu. Rev. Pathol..

[10] Kolset S.O., Reinholt F.P., Jenssen T. (2012). Diabetic Nephropathy and Extracellular Matrix. J. Histochem. Cytochem..

[11] Scindia Y.M., Deshmukh U.S., Bagavant H. (2010). Mesangial Pathology in Glomerular Disease: Targets for Therapeutic Intervention. Adv. Drug Delivery Rev..

[12] Schlöndorff D., Banas B. (2009). The Mesangial Cell Revisited: No Cell Is an Island. J. Am. Soc. Nephrol..

[13] Arora M.K., Singh U.K. (2013). Molecular mechanisms in the pathogenesis of diabetic nephropathy: An update. Vascul. Pharmacol..

[14] Huynh P., Chai Z. (2019). Transforming growth factor β (TGF-β) and related molecules in chronic kidney disease (CKD). Clin. Sci..

[15] Zeng L.-F., Xiao Y., Sun L. (2019). A Glimpse of the Mechanisms Related to Renal Fibrosis in Diabetic Nephropathy. Adv. Exp. Med. Biol..

[16] Ruggenenti P., Cravedi P., Remuzzi G. (2010). The RAAS in the pathogenesis and treatment of diabetic nephropathy. Nat. Rev. Nephrol..

[17] Giacco F., Brownlee M. (2010). Oxidative stress and diabetic complications. Circ. Res..

[18] Tessari P. (2015). Nitric oxide in the normal kidney and in patients with diabetic nephropathy. J. Nephrol..

[19] Schinner E., Wetzl V., Schlossmann J. (2015). Cyclic nucleotide signalling in kidney fibrosis. Int. J. Mol. Sci..

[20] Shen K., Johnson D.W., Gobe G.C. (2016). The role of cGMP and its signaling pathways in kidney disease. Am. J. Physiol. Renal Physiol..

[21] Krishnan S., Kraehling J., Eitner F., Bénardeau A., Sandner P. (2018). The Impact of the Nitric Oxide (NO)/Soluble Guanylyl Cyclase (sGC) Signaling Cascade on Kidney Health and Disease: A Preclinical Perspective. Int. J. Mol. Sci..

[22] Shi Y., Vanhoutte P.M. (2017). Macro- and microvascular endothelial dysfunction in diabetes. J. Diabetes.

[23] Hobbs A.J., Stasch J.-P. (2010). Soluble Guanylate Cyclase. Nitric Oxide: Biology and Pathobiology.

[24] Murad F. (2006). Nitric Oxide and Cyclic GMP in Cell Signaling and Drug Development. N. Engl. J. Med..

[25] Lundberg J.O., Gladwin M.T., Weitzberg E. (2015). Strategies to increase nitric oxide signalling in cardiovascular disease. Nat. Rev. Drug Discov..

[26] Mátyás C., Németh B.T., Oláh A., Hidi L., Birtalan E., Kellermayer D., Ruppert M., Korkmaz-Icöz S., Kökény G., Horváth E.M. (2015). The soluble guanylate cyclase activator cinaciguat prevents cardiac dysfunction in a rat model of type-1 diabetes mellitus. Cardiovasc. Diabetol..

[27] Kalk P., Godes M., Relle K., Rothkegel C., Hucke A., Stasch J.-P., Hocher B. (2006). NO-independent activation of soluble guanylate cyclase prevents disease progression in rats with 5/6 nephrectomy. Br. J. Pharmacol..

[28] Hoffmann L.S., Kretschmer A., Lawrenz B., Hocher B., Stasch J.-P. (2015). Chronic Activation of Heme Free Guanylate Cyclase Leads to Renal Protection in Dahl Salt-Sensitive Rats. PLoS ONE.

[29] Hohenstein B., Daniel C., Wagner A., Stasch J.-P., Hugo C. (2005). Stimulation of soluble guanylyl cyclase inhibits mesangial cell proliferation and matrix accumulation in experimental glomerulonephritis. Am. J. Physiol. Renal Physiol..

[30] Czirok S., Fang L., Radovits T., Szabó G., Szénási G., Rosivall L., Merkely B., Kökény G. (2017). Cinaciguat ameliorates glomerular damage by reducing ERK1/2 activity and TGF-ß expression in type-1 diabetic rats. Sci. Rep..

[31] Beyer C., Zenzmaier C., Palumbo-Zerr K., Mancuso R., Distler A., Dees C., Zerr P., Huang J., Maier C., Pachowsky M.L. (2015). Stimulation of the soluble guanylate cyclase (sGC) inhibits fibrosis by blocking non-canonical TGFβ signalling. Ann. Rheum. Dis..

[32] Erdmann E., Semigran M.J., Nieminen M.S., Gheorghiade M., Agrawal R., Mitrovic V., Mebazaa A. (2013). Cinaciguat, a soluble guanylate cyclase activator, unloads the heart but also causes hypotension in acute decompensated heart failure. Eur. Heart J..

[33] Glassman P.M., Muzykantov V.R. (2019). Pharmacokinetic and Pharmacodynamic Properties of Drug Delivery Systems. J. Pharmacol. Exp. Ther..

[34] Yoo J., Park C., Lee D., Koo H. (2019). Active Targeting Strategies Using Biological Ligands for Nanoparticle Drug Delivery Systems. Cancers.

[35] Nag O.K., Delehanty J.B. (2019). Active Cellular and Subcellular Targeting of Nanoparticles for Drug Delivery. Pharmaceutics.

[36] Wang K., Wen H.F., Yu D.G., Yang Y., Zhang D.F. (2018). Electrosprayed hydrophilic nanocomposites coated with shellac for colon-specific delayed drug delivery. Mater. Des..

[37] Colino C.I., Lanao J.M., Gutierrez-Milan C. (2020). Targeting of Hepatic Macrophages by Therapeutic Nanoparticles. Front. Immunol..

[38] Li S., Zeng Y., Peng K., Liu C., Zhang Z., Zhang L. (2019). Design and evaluation of glomerulus mesangium-targeted PEG-PLGA nanoparticles loaded with dexamethasone acetate. Acta Pharm. Sin..

[39] Fleischmann D., Maslanka Figueroa S., Beck S., Abstiens K., Witzgall R., Schweda F., Tauber P., Goepferich A. (2020). Adenovirus-Mimetic Nanoparticles: Sequential Ligand-Receptor Interplay as a Universal Tool for Enhanced In Vitro/In Vivo Cell Identification. ACS Appl. Mater. Interfaces.

[40] Maslanka Figueroa S., Fleischmann D., Goepferich A. (2020). Biomedical nanoparticle design: What we can learn from viruses. J. Control. Release.

[41] Fleischmann D., Maslanka Figueroa S., Goepferich A. (2020). Steric Shielding of cRGD-Functionalized Nanoparticles from Premature Exposition to Off-Target Endothelial Cells under a Physiological Flow. ACS Appl. Bio Mater..

[42] Maslanka Figueroa S., Fleischmann D., Beck S., Goepferich A. (2020). Thermodynamic, Spatial and Methodological Considerations for the Manufacturing of Therapeutic Polymer Nanoparticles. Pharm. Res..

[43] Thakral S., Thakral N.K. (2013). Prediction of drug-polymer miscibility through the use of solubility parameter based Flory-Huggins interaction parameter and the experimental validation: PEG as model polymer. J. Pharm. Sci..

[44] Satchell S.C., Braet F. (2009). Glomerular Endothelial Cell Fenestrations: An Integral Component of the Glomerular Filtration Barrier. Am. J. Physiol. Renal Physiol..

[45] Nam H.Y., Kwon S.M., Chung H., Lee S.-Y., Kwon S.-H., Jeon H., Kim Y., Park J.H., Kim J., Her S. (2009). Cellular uptake mechanism and intracellular fate of hydrophobically modified glycol chitosan nanoparticles. J. Control. Release.

[46] Seo S.-J., Chen M., Wang H., Kang M.S., Leong K.W., Kim H.-W. (2017). Extra- and intra-cellular fate of nanocarriers under dynamic interactions with biology. Nano Today.

[47] Meurer S., Pioch S., Pabst T., Opitz N., Schmidt P.M., Beckhaus T., Wagner K., Matt S., Gegenbauer K., Geschka S. (2009). Nitric oxide-independent vasodilator rescues heme-oxidized soluble guanylate cyclase from proteasomal degradation. Circ. Res..

[48] Evgenov O.V., Pacher P., Schmidt P.M., Hasko G., Schmidt H.H.H.W., Stasch J.-P. (2006). NO-independent stimulators and activators of soluble guanylate cyclase: Discovery and therapeutic potential. Nat. Rev. Drug Discov..

[49] Martin F., Baskaran P., Ma X., Dunten P.W., Schaefer M., Stasch J.-P., Beuve A., van den Akker F. (2010). Structure of cinaciguat (BAY 58–2667) bound to Nostoc H-NOX domain reveals insights into heme-mimetic activation of the soluble guanylyl cyclase. J. Biol. Chem..

[50] Smolenski A., Burckhardt M., Eigenthaler M., Butt E., Gambaryan S., Lohmann S.M., Walter U. (1998). Functional analysis of cGMP-dependent protein kinases I and II as mediators of NO/cGMP effects. Naunyn-Schmiedeberg’s Arch. Pharmacol..

[51] Geiselhöringer A., Gaisa M., Hofmann F., Schlossmann J. (2004). Distribution of IRAG and cGKI-isoforms in murine tissues. FEBS Lett..

[52] Kurtz A., Jelkmann W., Bauer C. (1982). Mesangial Cells Derived from Glomeruli Produce an Erythropoiesis Stimulating Factor in Cell Culture. FEBS Lett..

[53] Pfeilschifter J., Schalkwijk C., Briner V.A., van den Bosch H. (1993). Cytokine-stimulated secretion of group II phospholipase A2 by rat mesangial cells. Its contribution to arachidonic acid release and prostaglandin synthesis by cultured rat glomerular cells. J. Clin. Investig..

[54] Maslanka Figueroa S., Fleischmann D., Beck S., Tauber P., Witzgall R., Schweda F., Goepferich A. (2020). Nanoparticles Mimicking Viral Cell Recognition Strategies Are Superior Transporters into Mesangial Cells. Adv. Sci..

[55] Abstiens K., Gregoritza M., Goepferich A.M. (2019). Ligand Density and Linker Length are Critical Factors for Multivalent Nanoparticle-Receptor Interactions. ACS Appl. Mater. Interfaces.

[56] Maslanka Figueroa S., Veser A., Abstiens K., Fleischmann D., Beck S., Goepferich A. (2019). Influenza A Virus Mimetic Nanoparticles Trigger Selective Cell Uptake. Proc. Natl. Acad. Sci. USA.

[57] Muenster U., Becker-Pelster E.-M., Mao S., Ni R., Liang Z. (2016). Process for the Preparation of Porous Microparticles. U.S. Patent.

[58] Kansal A.R., Torquato S., Stillingser F.H. (2002). Computer generation of dense polydisperse sphere packings. J. Chem. Phys..

[59] Fedors R.F. (1974). A method for estimating both the solubility parameters and molar volumes of liquids. Polym. Eng. Sci..

[60] Schramm A., Mueller-Thuemen P., Littmann T., Harloff M., Ozawa T., Schlossmann J. (2018). Establishing a Split Luciferase Assay for Proteinkinase G (PKG) Interaction Studies. Int. J. Mol. Sci..

[61] Lowry O.H., Rosebrough N.J., Lewis Farr A., Randall R.J. (1951). Protein Measurement with the Folin Phenol Reagent. J. Biol. Chem..

[62] (2020). Image Lab 6.1 Software.

[63] Danielpour D., Kim K.Y., Dart L.L., Watanabe S., Roberts A.B., Sporn M. (1989). Sandwich Enzyme-Linked Immunosorbent Assays (Selisas) Quantitate and Distinguish Two Forms of Transforming Growth Factor-Beta (TGF-β1 and TGF-β2) in Complex Biological Fluids. Growth Factors.

[64] (2020). GraphPad Prism 6 Software.

