# Supplementary material for: Targeted Delivery of Soluble Guanylate Cyclase (sGC) Activator Cinaciguat to Renal Mesangial Cells via Virus-Mimetic Nanoparticles Potentiates Anti-Fibrotic Effects by cGMP-Mediated Suppression of the TGF-β Pathway
Source: Int J Mol Sci. 2021 Mar 4;22(5):2557. doi: 10.3390/ijms22052557 (PMC7961750; doi:10.3390/ijms22052557)
Supplement: Supplementary file 1 [file ijms-22-02557-s001.pdf]

Supporting information

Targeted Delivery of Soluble Guanylate Cyclase (sGC) Activator Cinaciguat to Renal Mesangial Cells *via* Virus-Mimetic Nanoparticles Potentiates Anti-Fibrotic Effects by cGMP-Mediated Suppression of the TGF-β Pathway

Daniel Fleischmann[1], Manuela Harloff[2], Sara Maslanka Figueroa[1], Jens Schlossmann[2], and Achim Goepferich[1*]

[1] Department of Pharmaceutical Technology, University of Regensburg, Universitaetsstrasse 31, 93053 Regensburg, Germany
[2] Department of Pharmacology and Toxicology, University of Regensburg, Universitaetsstrasse 31, 93053 Regensburg, Germany

*Corresponding author. E-mail: Achim.Goepferich@ur.de

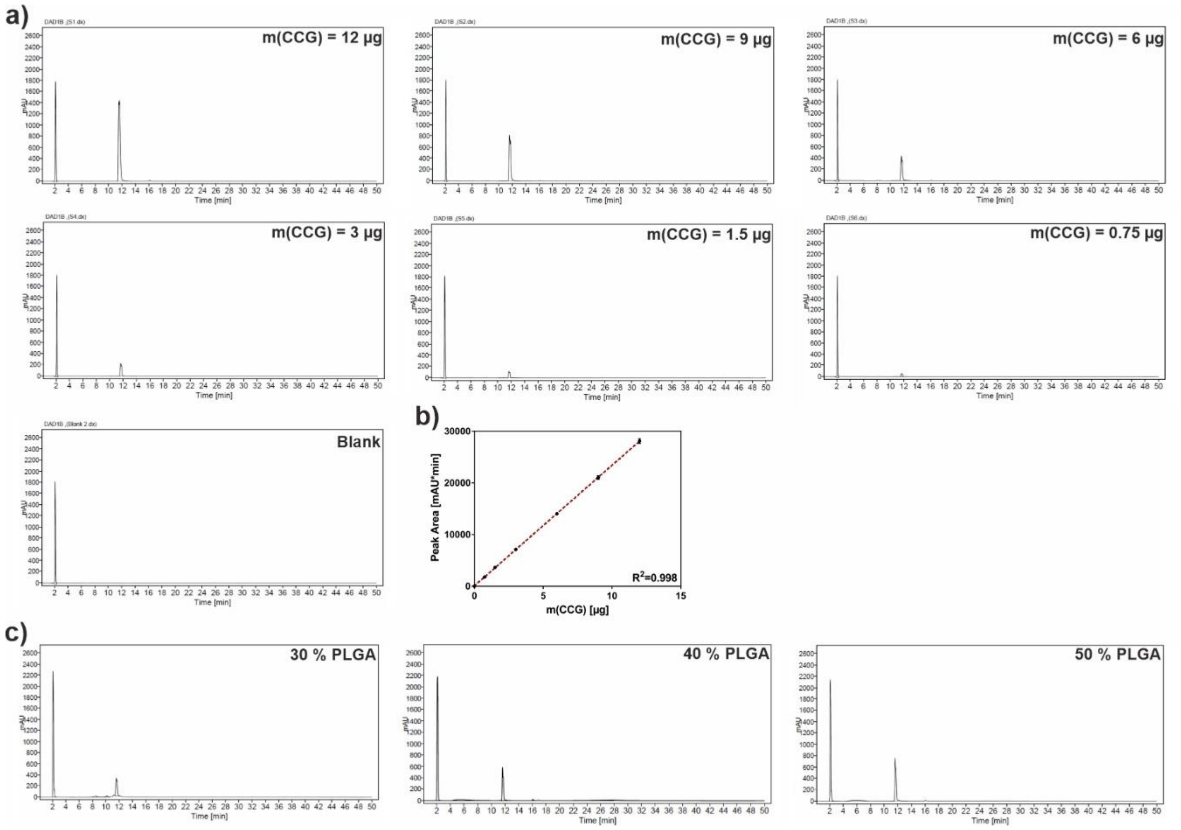

Figure S1. HPLC chromatograms. **a)** Representative HPLC chromatograms for the calibration of free Cinaciguat. **b)** HPLC calibration. Results represent mean ± SD (n = 3). **c)** Representative chromatograms for NP species carrying 30-50% PLGA and an initial CCG addition of 10 µg per 4 mg NP batch.

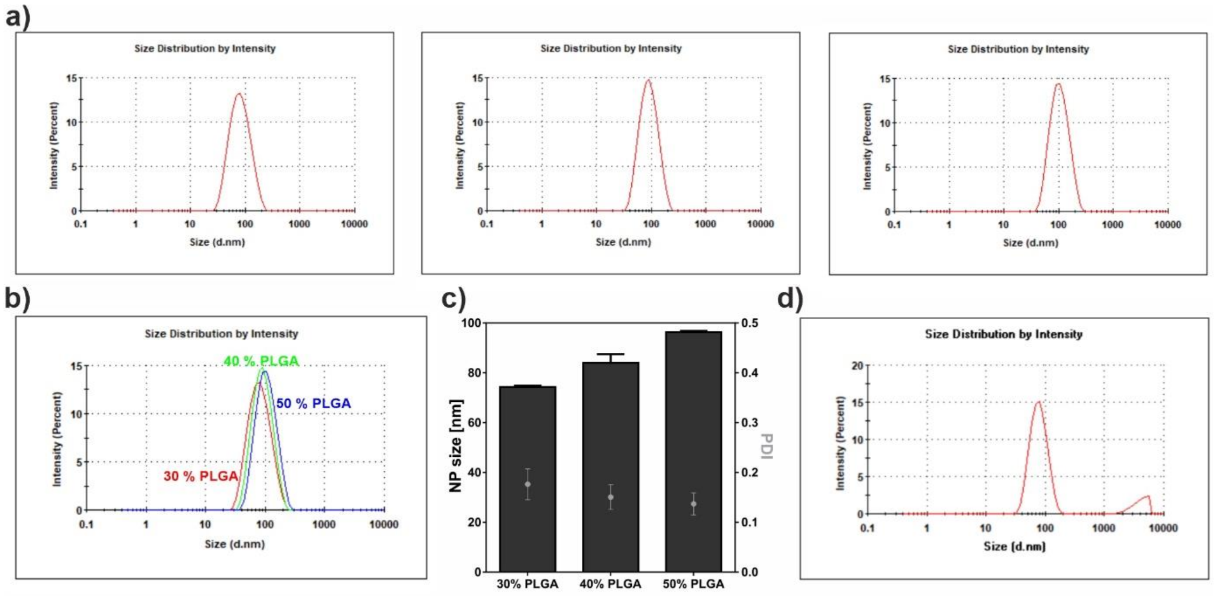

Figure S2. a) DLS size distribution of NP species containing 30/40/50% PLGA (from left to right). **b)** Merged DLS graphs. **c)** Size and polydispersity index (PDI) data for different NP species. **d)** Representative DLS graph of 40% PLGA NPs and an addition of 17.5 µg per 4 mg NP batch, showing a strong tendency for larger aggregates (see right peak).

a)

	size NP [nm]	d(NP core) [nm]	V(NP core) [nm^3]
30 % PLGA 1	75	11	697
30 % PLGA 2	74	10	523
30 % PLGA 3	74	10	523
40 % PLGA 1	82	18	3052
40 % PLGA 2	82	18	3052
40 % PLGA 3	88	19	3590
50 % PLGA 1	97	33	18807
50 % PLGA 2	96	32	17149
50 % PLGA 3	96	32	17149

b)

	size NP [nm]	m(CCG) [µg]	N(CCG)	m(NP) [mg]	n(NP) [mol]	N(NP)	CCG molecules/NP	CCG molecules/NP(ideal)	Entrapment efficiency [%]	Encapsulation efficiency [%]
30 % PLGA 1	75	2,59	2,76E+15	2,99	1,80E-11	1,08E+13	254,74	865,22	29,44	25,90
30 % PLGA 2	74	3,72	3,96E+15	3,45	2,16E-11	1,30E+13	304,88	650,05	46,90	37,24
30 % PLGA 3	74	3,69	3,93E+15	3,12	1,95E-11	1,18E+13	334,05	650,05	51,39	36,90
40 % PLGA 1	82	4,73	5,04E+15	3,14	1,44E-11	8,70E+12	579,07	3791,11	15,27	47,31
40 % PLGA 2	82	4,93	5,24E+15	3,02	1,39E-11	8,36E+12	626,80	3791,11	16,53	49,25
40 % PLGA 3	88	4,16	4,43E+15	3,02	1,32E-11	7,96E+12	556,32	4458,72	12,48	41,59
50 % PLGA 1	97	6,05	6,44E+15	3,11	8,64E-12	5,20E+12	1237,84	23360,97	5,30	60,51
50 % PLGA 2	96	5,66	6,03E+15	3,05	8,74E-12	5,26E+12	1145,17	21300,95	5,38	56,64
50 % PLGA 3	96	5,52	5,88E+15	2,98	8,54E-12	5,14E+12	1142,50	21300,95	5,36	55,21

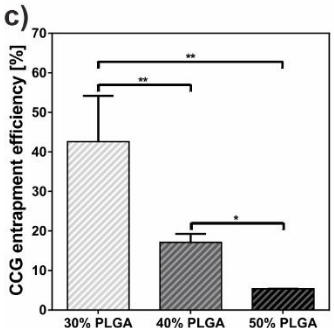

Figure S3. a) Results for calculation of NP core volume for each sample. **b)** Results for calculation of CCG encapsulation as well as entrapment efficiency. **c)** CCG entrapment efficiency per NP type.

Results represent mean ± SD (n = 3). *P<0.05 **P<0.01.

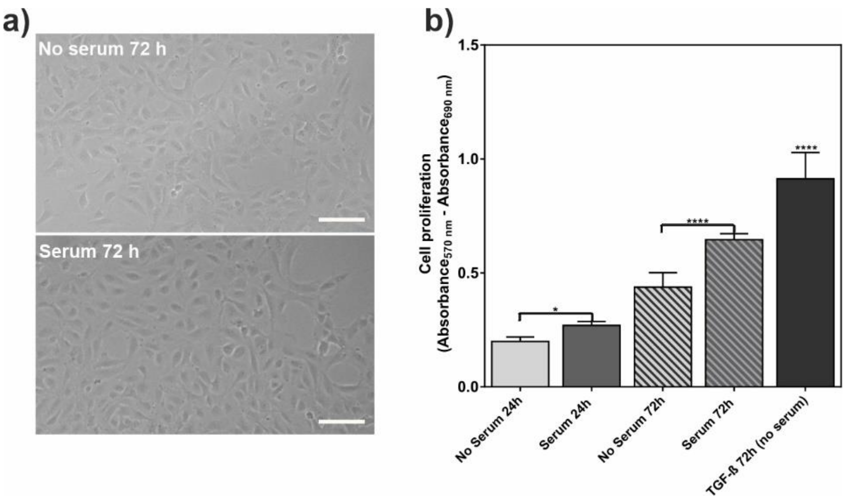

Figure S4. a) Brightfield images of rMCs cultured for 72 h during the cell proliferation assay either without (top) or with (bottom) addition of 10 % FBS. (Scale bar 100 µm). **b)** MTT assay results of rMC proliferation after 24 h and 72 h with and without addition of 10 % FBS. (Note: The results for samples “No serum 72 h” and “TGF-β 72 h (no serum)” represent the blank-normalized samples “Blank cells” and “TGF-β” in Figure 7a of the main manuscript.) Results represent mean ± SD (n = 3). *P<0.05 ****P<0.0001.
